# Supplementary material for: Vaccine-Related Autoimmune Hepatitis: Emerging Association with SARS-CoV-2 Vaccination or Coincidence?
Source: Vaccines (Basel). 2022 Dec 4;10(12):2073. doi: 10.3390/vaccines10122073 (PMC9783100; doi:10.3390/vaccines10122073)
Supplement: Supplementary file 1 [file vaccines-10-02073-s001.zip › Supplements/Supplement S1_Simplified Diagnostic Criteria for AIH.pdf]

Simplified Diagnostic Criteria for Autoimmune Hepatitis (AIH)

Probability
Definite AIH: ≥7
Probable AIH: ≥6

(Hennes EM, Zeniya M, Czaja AJ, Pares A, Dalekos GN, Krawitt EL, et. Simplified criteria for the diagnosis of autoimmune hepatitis. Hepatology. 2008; 48(1):169-76)

Variable	Cutoff	Points	Case by *Barary et al* (Points)	Total score
**ANA or SMA**	≥1:40	1	0	Score: 2<br>→ Non-probable AIH
**ANA or SMA**	≥1:80	2	0	
**or LKM**	≥1:40			
**or SLA**	Positive			
**IgG**	>Upper normal limit	1	0	
	>1.10 times upper normal limit	2		
**Liver histology** (evidence of hepatitis is a necessary condition)	Compatible with AIH	1	0	
	Typical AIH	2		
**Absence of viral hepatitis**	Yes	2	2	

AIH: Autoimmune hepatitis; ANA: antinuclear antibodies; LKM: liver-kidney microsomal antibodies; SLA: soluble liver antigen antibodies; SMA: smooth muscle antibodies;
*Addition of points achieved for all autoantibodies (maximum, 2 points).

Variable	Cutoff	Points	Case by *Mekritthikrai et al* (Points)	Total score
**ANA or SMA**	≥1:40	1	1	Score: 8<br>→ Definite AIH
**ANA or SMA**	≥1:80	2	2	
**or LKM**	≥1:40			
**or SLA**	Positive			
**IgG**	>Upper normal limit	1	1	
	>1.10 times upper normal limit	2		
**Liver histology** (evidence of hepatitis is a necessary condition)	Compatible with AIH	1	2	
	Typical AIH	2		
**Absence of viral hepatitis**	Yes	2	2	

AIH: Autoimmune hepatitis; ANA: antinuclear antibodies; LKM: liver-kidney microsomal antibodies; SLA: soluble liver antigen antibodies; SMA: smooth muscle antibodies;
*Addition of points achieved for all autoantibodies (maximum, 2 points).

Variable	Cutoff	Points	Case by *Brubaker et al* (Points)	Total score
**ANA or SMA**	≥1:40	1	0	Score: 4<br>→ Non-probable AIH
**ANA or SMA**	≥1:80	2	0	
**or LKM**	≥1:40			
**or SLA**	Positive			
**IgG**	>Upper normal limit	1	0	
	>1.10 times upper normal limit	2		
**Liver histology** (evidence of hepatitis is a necessary condition)	Compatible with AIH	1	2	
	Typical AIH	2		
**Absence of viral hepatitis**	Yes	2	2	

AIH: Autoimmune hepatitis; ANA: antinuclear antibodies; LKM: liver-kidney microsomal antibodies; SLA: soluble liver antigen antibodies; SMA: smooth muscle antibodies;
*Addition of points achieved for all autoantibodies (maximum, 2 points).

Variable	Cutoff	Points	Case 1 by *Shahrani et al* (Points)	Total score
**ANA or SMA**	≥1:40	1	0	Score: 4<br>→ Non-probable AIH
**ANA or SMA**	≥1:80	2	0	
**or LKM**	≥1:40			
**or SLA**	Positive			
**IgG**	>Upper normal limit	1	1	
	>1.10 times upper normal limit	2		
**Liver histology** (evidence of hepatitis is a necessary condition)	Compatible with AIH	1	1	
	Typical AIH	2		
**Absence of viral hepatitis**	Yes	2	2	

AIH: Autoimmune hepatitis; ANA: antinuclear antibodies; LKM: liver-kidney microsomal antibodies; SLA: soluble liver antigen antibodies; SMA: smooth muscle antibodies;
*Addition of points achieved for all autoantibodies (maximum, 2 points).

Variable	Cutoff	Points	Case 2 by *Shahrani et al* (Points)	Total score
**ANA or SMA**	≥1:40	1	1	Score: 6<br>→ Probable AIH
**ANA or SMA**	≥1:80	2	0	
**or LKM**	≥1:40			
**or SLA**	Positive			
**IgG**	>Upper normal limit	1	1	
	>1.10 times upper normal limit	2		
**Liver histology** (evidence of hepatitis is a necessary condition)	Compatible with AIH	1	2	
	Typical AIH	2		
**Absence of viral hepatitis**	Yes	2	2	

AIH: Autoimmune hepatitis; ANA: antinuclear antibodies; LKM: liver-kidney microsomal antibodies; SLA: soluble liver antigen antibodies; SMA: smooth muscle antibodies;
*Addition of points achieved for all autoantibodies (maximum, 2 points).

Variable	Cutoff	Points	Case 3 by *Shahrani et al* (Points)	Total score
**ANA or SMA**	≥1:40	1	1	Score: 5<br>→ Non-probable AIH
**ANA or SMA**	≥1:80	2	0	
**or LKM**	≥1:40			
**or SLA**	Positive			
**IgG**	>Upper normal limit	1	1	
	>1.10 times upper normal limit	2		
**Liver histology** (evidence of hepatitis is a necessary condition)	Compatible with AIH	1	1	
	Typical AIH	2		
**Absence of viral hepatitis**	Yes	2	2	

AIH: Autoimmune hepatitis; ANA: antinuclear antibodies; LKM: liver-kidney microsomal antibodies; SLA: soluble liver antigen antibodies; SMA: smooth muscle antibodies;
*Addition of points achieved for all autoantibodies (maximum, 2 points).

Variable	Cutoff	Points	Case by *Hasegawa et al* (Points)	Total score
**ANA or SMA**	≥1:40	1	1	
**ANA or SMA**	≥1:80	2	0	
**or LKM**	≥1:40			
**or SLA**	Positive			
**IgG**	>Upper normal limit	1	1	**Score: 6**<br>**→ Probable AIH**
	>1.10 times upper normal limit	2		
**Liver histology** (evidence of hepatitis is a necessary condition)	Compatible with AIH	1	2	
	Typical AIH	2		
**Absence of viral hepatitis**	Yes	2	2	

AIH: Autoimmune hepatitis; ANA: antinuclear antibodies; LKM: liver-kidney microsomal antibodies; SLA: soluble liver antigen antibodies; SMA: smooth muscle antibodies;
*Addition of points achieved for all autoantibodies (maximum, 2 points).

Variable	Cutoff	Points	Case by *Lasagna et al* (Points)	Total score
**ANA or SMA**	≥1:40	1	0	
**ANA or SMA**	≥1:80	2	0	
**or LKM**	≥1:40			
**or SLA**	Positive			
**IgG**	>Upper normal limit	1	0	**Score: 0**<br>**→ Non-probable AIH**
	>1.10 times upper normal limit	2		
**Liver histology** (evidence of hepatitis is a necessary condition)	Compatible with AIH	1	0	
	Typical AIH	2		
**Absence of viral hepatitis**	Yes	2	0	

AIH: Autoimmune hepatitis; ANA: antinuclear antibodies; LKM: liver-kidney microsomal antibodies; SLA: soluble liver antigen antibodies; SMA: smooth muscle antibodies;
*Addition of points achieved for all autoantibodies (maximum, 2 points).

Variable	Cutoff	Points	Case 1 by *Pinazo-Bandera et al* (Points)	Total score
**ANA or SMA**	≥1:40	1	0	
**ANA or SMA**	≥1:80	2	2	
**or LKM**	≥1:40			
**or SLA**	Positive			
**IgG**	>Upper normal limit	1	0	**Score: 6**<br>**→ Probable AIH**
	>1.10 times upper normal limit	2		
**Liver histology** (evidence of hepatitis is a necessary condition)	Compatible with AIH	1	2	
	Typical AIH	2		
**Absence of viral hepatitis**	Yes	2	2	

AIH: Autoimmune hepatitis; ANA: antinuclear antibodies; LKM: liver-kidney microsomal antibodies; SLA: soluble liver antigen antibodies; SMA: smooth muscle antibodies;
*Addition of points achieved for all autoantibodies (maximum, 2 points).

Variable	Cutoff	Points	Case 2 by *Pinazo-Bandera et al* (Points)	Total score
**ANA or SMA**	≥1:40	1	0	Score: 4<br><br>→ Non-probable AIH
**ANA or SMA**	≥1:80	2	0	
**or LKM**	≥1:40			
**or SLA**	Positive			
**IgG**	>Upper normal limit	1	0	
	>1.10 times upper normal limit	2		
**Liver histology** (evidence of hepatitis is a necessary condition)	Compatible with AIH	1	2	
	Typical AIH	2		
**Absence of viral hepatitis**	Yes	2	2	

AIH: Autoimmune hepatitis; ANA: antinuclear antibodies; LKM: liver-kidney microsomal antibodies; SLA: soluble liver antigen antibodies; SMA: smooth muscle antibodies;
*Addition of points achieved for all autoantibodies (maximum, 2 points).

Variable	Cutoff	Points	Case by *Boettler et al* (Points)	Total score
**ANA or SMA**	≥1:40	1	0	Score: 7<br><br>→ Definite AIH
**ANA or SMA**	≥1:80	2	2	
**or LKM**	≥1:40			
**or SLA**	Positive			
**IgG**	>Upper normal limit	1	1	
	>1.10 times upper normal limit	2		
**Liver histology** (evidence of hepatitis is a necessary condition)	Compatible with AIH	1	2	
	Typical AIH	2		
**Absence of viral hepatitis**	Yes	2	2	

AIH: Autoimmune hepatitis; ANA: antinuclear antibodies; LKM: liver-kidney microsomal antibodies; SLA: soluble liver antigen antibodies; SMA: smooth muscle antibodies;
*Addition of points achieved for all autoantibodies (maximum, 2 points).

Variable	Cutoff	Points	Case by *Kang et al* (Points)	Total score
**ANA or SMA**	≥1:40	1	0	Score: 7<br><br>→ Definite AIH
**ANA or SMA**	≥1:80	2	2	
**or LKM**	≥1:40			
**or SLA**	Positive			
**IgG**	>Upper normal limit	1	1	
	>1.10 times upper normal limit	2		
**Liver histology** (evidence of hepatitis is a necessary condition)	Compatible with AIH	1	2	
	Typical AIH	2		
**Absence of viral hepatitis**	Yes	2	2	

AIH: Autoimmune hepatitis; ANA: antinuclear antibodies; LKM: liver-kidney microsomal antibodies; SLA: soluble liver antigen antibodies; SMA: smooth muscle antibodies;
*Addition of points achieved for all autoantibodies (maximum, 2 points).

Variable	Cutoff	Points	Case by *Ghorbani et al* (Points)	Total score
**ANA or SMA**	≥1:40	1	0	
**ANA or SMA**	≥1:80	2	0	
**or LKM**	≥1:40			
**or SLA**	Positive			
**IgG**	>Upper normal limit	1	0	**Score: 3**<br>**→ Non-probable AIH**
	>1.10 times upper normal limit	2		
**Liver histology** (evidence of hepatitis is a necessary condition)	Compatible with AIH	1	1	
	Typical AIH	2		
**Absence of viral hepatitis**	Yes	2	2	

AIH: Autoimmune hepatitis; ANA: antinuclear antibodies; LKM: liver-kidney microsomal antibodies; SLA: soluble liver antigen antibodies; SMA: smooth muscle antibodies;
*Addition of points achieved for all autoantibodies (maximum, 2 points).

Variable	Cutoff	Points	Case by *Fimiano et al* (Points)	Total score
**ANA or SMA**	≥1:40	1	0	
**ANA or SMA**	≥1:80	2	0	
**or LKM**	≥1:40			
**or SLA**	Positive			
**IgG**	>Upper normal limit	1	1	**Score: 3**<br>**→ Non-probable AIH**
	>1.10 times upper normal limit	2		
**Liver histology** (evidence of hepatitis is a necessary condition)	Compatible with AIH	1	2	
	Typical AIH	2		
**Absence of viral hepatitis**	Yes	2	2	

AIH: Autoimmune hepatitis; ANA: antinuclear antibodies; LKM: liver-kidney microsomal antibodies; SLA: soluble liver antigen antibodies; SMA: smooth muscle antibodies;
*Addition of points achieved for all autoantibodies (maximum, 2 points).

Variable	Cutoff	Points	Case by *Camacho-Dominguez et al* (Points)	Total score
**ANA or SMA**	≥1:40	1	0	
**ANA or SMA**	≥1:80	2	2	
**or LKM**	≥1:40			
**or SLA**	Positive			
**IgG**	>Upper normal limit	1	1	**Score: 3**<br>**→ Non-probable AIH**
	>1.10 times upper normal limit	2		
**Liver histology** (evidence of hepatitis is a necessary condition)	Compatible with AIH	1	2	
	Typical AIH	2		
**Absence of viral hepatitis**	Yes	2	2	

AIH: Autoimmune hepatitis; ANA: antinuclear antibodies; LKM: liver-kidney microsomal antibodies; SLA: soluble liver antigen antibodies; SMA: smooth muscle antibodies;
*Addition of points achieved for all autoantibodies (maximum, 2 points).

Variable	Cutoff	Points	Cases by *Erard et al* (Points)	Total score
**ANA or SMA**	≥1:40	1	Values are not available. At least one point for ANA positivity on each patient.	**Score≥ 6 for each patient** **→ At least probable AIH**
**ANA or SMA**	≥1:80	2		
**or LKM**	≥1:40			
**or SLA**	Positive			
**IgG**	>Upper normal limit	1	Values are not available. At least one point for IgG increase on each patient.	
	>1.10 times upper normal limit	2		
**Liver histology** (evidence of hepatitis is a necessary condition)	Compatible with AIH	1	All three patients had liver biopsy typical for AIH. Two points for each.	
	Typical AIH	2		
**Absence of viral hepatitis**	Yes	2	All three patients were tested negative for viral hepatitis. Two points for each.	

AIH: Autoimmune hepatitis; ANA: antinuclear antibodies; LKM: liver-kidney microsomal antibodies; SLA: soluble liver antigen antibodies; SMA: smooth muscle antibodies;
*Addition of points achieved for all autoantibodies (maximum, 2 points).

Variable	Cutoff	Points	Case 1 by *Suzuki et al* (Points)	Total score
**ANA or SMA**	≥1:40	1	1	**Score: 6** **→ Probable AIH**
**ANA or SMA**	≥1:80	2	0	
**or LKM**	≥1:40			
**or SLA**	Positive			
**IgG**	>Upper normal limit	1	1	
	>1.10 times upper normal limit	2		
**Liver histology** (evidence of hepatitis is a necessary condition)	Compatible with AIH	1	2	
	Typical AIH	2		
**Absence of viral hepatitis**	Yes	2	2	

AIH: Autoimmune hepatitis; ANA: antinuclear antibodies; LKM: liver-kidney microsomal antibodies; SLA: soluble liver antigen antibodies; SMA: smooth muscle antibodies;
*Addition of points achieved for all autoantibodies (maximum, 2 points).

Variable	Cutoff	Points	Case 2 by *Suzuki et al* (Points)	Total score
**ANA or SMA**	≥1:40	1	0	**Score: 7** **→ Definite AIH**
**ANA or SMA**	≥1:80	2	2	
**or LKM**	≥1:40			
**or SLA**	Positive			
**IgG**	>Upper normal limit	1	1	
	>1.10 times upper normal limit	2		
**Liver histology** (evidence of hepatitis is a necessary condition)	Compatible with AIH	1	2	
	Typical AIH	2		
**Absence of viral hepatitis**	Yes	2	2	

AIH: Autoimmune hepatitis; ANA: antinuclear antibodies; LKM: liver-kidney microsomal antibodies; SLA: soluble liver antigen antibodies; SMA: smooth muscle antibodies;
*Addition of points achieved for all autoantibodies (maximum, 2 points).

Variable	Cutoff	Points	Case 3 by *Suzuki et al* (Points)	Total score
**ANA or SMA**	≥1:40	1	0	**Score: 7**<br>**→ Definite AIH**
**ANA or SMA**	≥1:80	2	2	
**or LKM**	≥1:40			
**or SLA**	Positive			
**IgG**	>Upper normal limit	1	1	
	>1.10 times upper normal limit	2		
**Liver histology** (evidence of hepatitis is a necessary condition)	Compatible with AIH	1	2	
	Typical AIH	2		
**Absence of viral hepatitis**	Yes	2	2	

AIH: Autoimmune hepatitis; ANA: antinuclear antibodies; LKM: liver-kidney microsomal antibodies; SLA: soluble liver antigen antibodies; SMA: smooth muscle antibodies;
*Addition of points achieved for all autoantibodies (maximum, 2 points).

Variable	Cutoff	Points	Case by *Palla et al* (Points)	Total score
**ANA or SMA**	≥1:40	1	0	**Score: 8**<br>**→ Definite AIH**
**ANA or SMA**	≥1:80	2	2	
**or LKM**	≥1:40			
**or SLA**	Positive			
**IgG**	>Upper normal limit	1	2	
	>1.10 times upper normal limit	2		
**Liver histology** (evidence of hepatitis is a necessary condition)	Compatible with AIH	1	2	
	Typical AIH	2		
**Absence of viral hepatitis**	Yes	2	2	

AIH: Autoimmune hepatitis; ANA: antinuclear antibodies; LKM: liver-kidney microsomal antibodies; SLA: soluble liver antigen antibodies; SMA: smooth muscle antibodies;
*Addition of points achieved for all autoantibodies (maximum, 2 points).

Variable	Cutoff	Points	Case by *Zhou et al* (Points)	Total score
**ANA or SMA**	≥1:40	1	0	**Score: 6**<br>**→ Probable AIH**
**ANA or SMA**	≥1:80	2	2	
**or LKM**	≥1:40			
**or SLA**	Positive			
**IgG**	>Upper normal limit	1	na	
	>1.10 times upper normal limit	2		
**Liver histology** (evidence of hepatitis is a necessary condition)	Compatible with AIH	1	2	
	Typical AIH	2		
**Absence of viral hepatitis**	Yes	2	2	

AIH: Autoimmune hepatitis; ANA: antinuclear antibodies; LKM: liver-kidney microsomal antibodies; SLA: soluble liver antigen antibodies; SMA: smooth muscle antibodies;
*Addition of points achieved for all autoantibodies (maximum, 2 points).

Variable	Cutoff	Points	Case by *Ghielmetti et al* (Points)	Total score
**ANA or SMA**	≥1:40	1	0	
**ANA or SMA**	≥1:80	2	2	
**or LKM**	≥1:40			
**or SLA**	Positive			
**IgG**	>Upper normal limit	1	1	**Score: 6**
	>1.10 times upper normal limit	2		**→ Probable AIH**
**Liver histology** (evidence of hepatitis is a necessary condition)	Compatible with AIH	1	2	
	Typical AIH	2		
**Absence of viral hepatitis**	Yes	2	2	

AIH: Autoimmune hepatitis; ANA: antinuclear antibodies; LKM: liver-kidney microsomal antibodies; SLA: soluble liver antigen antibodies; SMA: smooth muscle antibodies;
*Addition of points achieved for all autoantibodies (maximum, 2 points).

Variable	Cutoff	Points	Case by *McShane et al* (Points)	Total score
**ANA or SMA**	≥1:40	1	0	
**ANA or SMA**	≥1:80	2	2	
**or LKM**	≥1:40			
**or SLA**	Positive			
**IgG**	>Upper normal limit	1	1	
	>1.10 times upper normal limit	2		**Score: 7**
**Liver histology** (evidence of hepatitis is a necessary condition)	Compatible with AIH	1	2	**→ Definite AIH**
	Typical AIH	2		
**Absence of viral hepatitis**	Yes	2	2	

AIH: Autoimmune hepatitis; ANA: antinuclear antibodies; LKM: liver-kidney microsomal antibodies; SLA: soluble liver antigen antibodies; SMA: smooth muscle antibodies;
*Addition of points achieved for all autoantibodies (maximum, 2 points).

Variable	Cutoff	Points	Case by *Clayton-Chubb et al* (Points)	Total score
**ANA or SMA**	≥1:40	1	0	
**ANA or SMA**	≥1:80	2	2	
**or LKM**	≥1:40			
**or SLA**	Positive			
**IgG**	>Upper normal limit	1	0	**Score: 6**
	>1.10 times upper normal limit	2		**→ Probable AIH**
**Liver histology** (evidence of hepatitis is a necessary condition)	Compatible with AIH	1	2	
	Typical AIH	2		
**Absence of viral hepatitis**	Yes	2	2	

AIH: Autoimmune hepatitis; ANA: antinuclear antibodies; LKM: liver-kidney microsomal antibodies; SLA: soluble liver antigen antibodies; SMA: smooth muscle antibodies;
*Addition of points achieved for all autoantibodies (maximum, 2 points).

Variable	Cutoff	Points	Case by *Tan et al* (Points)	Total score
**ANA or SMA**	≥1:40	1	Values are not available. At least one point as the patient was ANA and ASMA positive.	Score ≥7<br>→ Definite AIH
**ANA or SMA**	≥1:80	2		
**or LKM**	≥1:40			
**or SLA**	Positive			
**IgG**	>Upper normal limit	1	2	
	>1.10 times upper normal limit	2		
**Liver histology** (evidence of hepatitis is a necessary condition)	Compatible with AIH	1	2	
	Typical AIH	2		
**Absence of viral hepatitis**	Yes	2	2	

AIH: Autoimmune hepatitis; ANA: antinuclear antibodies; LKM: liver-kidney microsomal antibodies; SLA: soluble liver antigen antibodies; SMA: smooth muscle antibodies;
*Addition of points achieved for all autoantibodies (maximum, 2 points).

Variable	Cutoff	Points	Case by *Rocco et al* (Points)	Total score
**ANA or SMA**	≥1:40	1	0	Score: 8<br>→ Definite AIH
**ANA or SMA**	≥1:80	2	2	
**or LKM**	≥1:40			
**or SLA**	Positive			
**IgG**	>Upper normal limit	1	2	
	>1.10 times upper normal limit	2		
**Liver histology** (evidence of hepatitis is a necessary condition)	Compatible with AIH	1	2	
	Typical AIH	2		
**Absence of viral hepatitis**	Yes	2	2	

AIH: Autoimmune hepatitis; ANA: antinuclear antibodies; LKM: liver-kidney microsomal antibodies; SLA: soluble liver antigen antibodies; SMA: smooth muscle antibodies;
*Addition of points achieved for all autoantibodies (maximum, 2 points).

Variable	Cutoff	Points	Case by *Bril et al* (Points)	Total score
**ANA or SMA**	≥1:40	1	0	Score: 6<br>→ Probable AIH
**ANA or SMA**	≥1:80	2	2	
**or LKM**	≥1:40			
**or SLA**	Positive			
**IgG**	>Upper normal limit	1	0	
	>1.10 times upper normal limit	2		
**Liver histology** (evidence of hepatitis is a necessary condition)	Compatible with AIH	1	2	
	Typical AIH	2		
**Absence of viral hepatitis**	Yes	2	2	

AIH: Autoimmune hepatitis; ANA: antinuclear antibodies; LKM: liver-kidney microsomal antibodies; SLA: soluble liver antigen antibodies; SMA: smooth muscle antibodies;
*Addition of points achieved for all autoantibodies (maximum, 2 points).

Variable	Cutoff	Points	Case by *Goulas et al* (Points)	Total score
**ANA or SMA**	≥1:40	1	0	
**ANA or SMA**	≥1:80	2	2	
**or LKM**	≥1:40			
**or SLA**	Positive			
**IgG**	>Upper normal limit	1	1	**Score: 7**
	>1.10 times upper normal limit	2		**→ Definite AIH**
**Liver histology** (evidence of hepatitis is a necessary condition)	Compatible with AIH	1	2	
	Typical AIH	2		
**Absence of viral hepatitis**	Yes	2	2	

AIH: Autoimmune hepatitis; ANA: antinuclear antibodies; LKM: liver-kidney microsomal antibodies; SLA: soluble liver antigen antibodies; SMA: smooth muscle antibodies;
*Addition of points achieved for all autoantibodies (maximum, 2 points).

Variable	Cutoff	Points	Case by *Lodato et al* (Points)	Total score
**ANA or SMA**	≥1:40	1	0	
**ANA or SMA**	≥1:80	2	0	
**or LKM**	≥1:40			
**or SLA**	Positive			
**IgG**	>Upper normal limit	1	0	**Score: 4**
	>1.10 times upper normal limit	2		**→ Non-probable AIH**
**Liver histology** (evidence of hepatitis is a necessary condition)	Compatible with AIH	1	2	
	Typical AIH	2		
**Absence of viral hepatitis**	Yes	2	2	

AIH: Autoimmune hepatitis; ANA: antinuclear antibodies; LKM: liver-kidney microsomal antibodies; SLA: soluble liver antigen antibodies; SMA: smooth muscle antibodies;
*Addition of points achieved for all autoantibodies (maximum, 2 points).

Variable	Cutoff	Points	Case 1 by *Rela et al* (Points)	Total score
**ANA or SMA**	≥1:40	1	0	
**ANA or SMA**	≥1:80	2	2	
**or LKM**	≥1:40			
**or SLA**	Positive			
**IgG**	>Upper normal limit	1	1	**Score: 7**
	>1.10 times upper normal limit	2		**→ Definite AIH**
**Liver histology** (evidence of hepatitis is a necessary condition)	Compatible with AIH	1	2	
	Typical AIH	2		
**Absence of viral hepatitis**	Yes	2	2	

AIH: Autoimmune hepatitis; ANA: antinuclear antibodies; LKM: liver-kidney microsomal antibodies; SLA: soluble liver antigen antibodies; SMA: smooth muscle antibodies;
*Addition of points achieved for all autoantibodies (maximum, 2 points).

Variable	Cutoff	Points	Case 2 by *Rela et al* (Points)	Total score
**ANA or SMA**	≥1:40	1	0	**Score: 3**<br><br>**→ Non-probable AIH**
**ANA or SMA**	≥1:80	2	0	
**or LKM**	≥1:40			
**or SLA**	Positive			
**IgG**	>Upper normal limit	1	0	
	>1.10 times upper normal limit	2		
**Liver histology** (evidence of hepatitis is a necessary condition)	Compatible with AIH	1	1	
	Typical AIH	2		
**Absence of viral hepatitis**	Yes	2	2	

AIH: Autoimmune hepatitis; ANA: antinuclear antibodies; LKM: liver-kidney microsomal antibodies; SLA: soluble liver antigen antibodies; SMA: smooth muscle antibodies;
*Addition of points achieved for all autoantibodies (maximum, 2 points).

Variable	Cutoff	Points	Case 2 by *Zin Tun et al* (Points)	Total score
**ANA or SMA**	≥1:40	1	No exact values are provided. At least one point as the patient was ANA positive.	**Score≥ 6**<br><br>**→ At least probable AIH**
**ANA or SMA**	≥1:80	2		
**or LKM**	≥1:40			
**or SLA**	Positive			
**IgG**	>Upper normal limit	1	1	
	>1.10 times upper normal limit	2		
**Liver histology** (evidence of hepatitis is a necessary condition)	Compatible with AIH	1	2	
	Typical AIH	2		
**Absence of viral hepatitis**	Yes	2	2	

AIH: Autoimmune hepatitis; ANA: antinuclear antibodies; LKM: liver-kidney microsomal antibodies; SLA: soluble liver antigen antibodies; SMA: smooth muscle antibodies;
*Addition of points achieved for all autoantibodies (maximum, 2 points).

Variable	Cutoff	Points	Case 1 by *Ferronato et al* (Points)	Total score
**ANA or SMA**	≥1:40	1	0	**Score: 7**<br><br>**→ Definite AIH**
**ANA or SMA**	≥1:80	2	2	
**or LKM**	≥1:40			
**or SLA**	Positive			
**IgG**	>Upper normal limit	1	1	
	>1.10 times upper normal limit	2		
**Liver histology** (evidence of hepatitis is a necessary condition)	Compatible with AIH	1	2	
	Typical AIH	2		
**Absence of viral hepatitis**	Yes	2	2	

AIH: Autoimmune hepatitis; ANA: antinuclear antibodies; LKM: liver-kidney microsomal antibodies; SLA: soluble liver antigen antibodies; SMA: smooth muscle antibodies;
*Addition of points achieved for all autoantibodies (maximum, 2 points).

Variable	Cutoff	Points	Case 2 by *Ferronato et al* (Points)	Total score
**ANA or SMA**	≥1:40	1	0	**Score: 7**<br>**→ Definite AIH**
**ANA or SMA**	≥1:80	2	2	
**or LKM**	≥1:40			
**or SLA**	Positive			
**IgG**	>Upper normal limit	1	1	
	>1.10 times upper normal limit	2		
**Liver histology** (evidence of hepatitis is a necessary condition)	Compatible with AIH	1	2	
	Typical AIH	2		
**Absence of viral hepatitis**	Yes	2	2	

AIH: Autoimmune hepatitis; ANA: antinuclear antibodies; LKM: liver-kidney microsomal antibodies; SLA: soluble liver antigen antibodies; SMA: smooth muscle antibodies;
*Addition of points achieved for all autoantibodies (maximum, 2 points).

Variable	Cutoff	Points	Case 3 by *Ferronato et al* (Points)	Total score
**ANA or SMA**	≥1:40	1	0	**Score: 7**<br>**→ Definite AIH**
**ANA or SMA**	≥1:80	2	2	
**or LKM**	≥1:40			
**or SLA**	Positive			
**IgG**	>Upper normal limit	1	0	
	>1.10 times upper normal limit	2		
**Liver histology** (evidence of hepatitis is a necessary condition)	Compatible with AIH	1	2	
	Typical AIH	2		
**Absence of viral hepatitis**	Yes	2	2	

AIH: Autoimmune hepatitis; ANA: antinuclear antibodies; LKM: liver-kidney microsomal antibodies; SLA: soluble liver antigen antibodies; SMA: smooth muscle antibodies;
*Addition of points achieved for all autoantibodies (maximum, 2 points).

Variable	Cutoff	Points	Case by *Mahalingham et al* (Points)	Total score
**ANA or SMA**	≥1:40	1	0	**Score: 6**<br>**→ Probable AIH**
**ANA or SMA**	≥1:80	2	2	
**or LKM**	≥1:40			
**or SLA**	Positive			
**IgG**	>Upper normal limit	1	0	
	>1.10 times upper normal limit	2		
**Liver histology** (evidence of hepatitis is a necessary condition)	Compatible with AIH	1	2	
	Typical AIH	2		
**Absence of viral hepatitis**	Yes	2	2	

AIH: Autoimmune hepatitis; ANA: antinuclear antibodies; LKM: liver-kidney microsomal antibodies; SLA: soluble liver antigen antibodies; SMA: smooth muscle antibodies;
*Addition of points achieved for all autoantibodies (maximum, 2 points).

Variable	Cutoff	Points	Case by *Lee et al* (Points)	Total score
**ANA or SMA**	≥1:40	1	0	**Score: 6**<br><br>**→ Probable AIH**
**ANA or SMA**	≥1:80	2	2	
**or LKM**	≥1:40			
**or SLA**	Positive			
**IgG**	>Upper normal limit	1	0	
	>1.10 times upper normal limit	2		
**Liver histology** (evidence of hepatitis is a necessary condition)	Compatible with AIH	1	2	
	Typical AIH	2		
**Absence of viral hepatitis**	Yes	2	2	

AIH: Autoimmune hepatitis; ANA: antinuclear antibodies; LKM: liver-kidney microsomal antibodies; SLA: soluble liver antigen antibodies; SMA: smooth muscle antibodies;
*Addition of points achieved for all autoantibodies (maximum, 2 points).
