# Supplementary material for: Vaccine-Related Autoimmune Hepatitis: Emerging Association with SARS-CoV-2 Vaccination or Coincidence?
Source: Vaccines (Basel). 2022 Dec 4;10(12):2073. doi: 10.3390/vaccines10122073 (PMC9783100; doi:10.3390/vaccines10122073)
Supplement: Supplementary file 1 [file vaccines-10-02073-s001.zip › Supplements/Supplement S2_Naranjo scale for ADRs.pdf]

Naranjo Nomogram for Adverse Drug Reaction Assessment

<u>Probability</u>
Definite: Score≥9
Probable: 5-8
Possible: 1-4
Doubtful: ≤0

(Michel DJ, Knobel Lc. Comparison of three algorithms used to evaluate adverse drug reactions. Am J Hosp Pharm. 1986; 43:1711)

**Case 1 by:** ***Torrente et al***	Yes	No	Do not know	Score	Comment
1. Are there previous conclusive reports on this reaction?	+1	0	0	1	≥2 published reports in which the adverse reaction has been described in detail
2. Did the adverse event appear after the suspected drug was administered?	+2	-1	0	2	Adverse reaction appeared soon after vaccination
3. Did the adverse reaction improve when the drug was discontinued, or a specific antagonist was administered?	+1	0	0	1	Rapid improvement after corticosteroids and azathioprine administration
4. Did the adverse reaction reappear when the drug was readministered?	+2	-1	0	0	
5. Are there alternative causes (other than the drug) that could on their own have caused the reaction?	-1	+2	0	0	
6. Did the reaction appeared when a placebo was given?	-1	+1	0	0	
7. Was the drug detected in the blood (or other body fluids) in concentrations known to be toxic?	+1	0	0	0	
8. Was the reaction more severe when the dose was increased, or less severe when dose decreased?	+1	0	0	0	
9. Did the patient have a similar reaction to the same or similar drugs in any previous exposure?	+1	0	0	0	
10. Was the adverse event confirmed by any objective evidence?	+1	0	0	1	Biopsy conformation of AIH

Total score: **5→ Probable adverse drug reaction**

**Case 2, 3, 4, 5 by:** ***Izagirre et al***	Yes	No	Do not know	Score	Comment
1. Are there previous conclusive reports on this reaction?	+1	0	0	1	≥2 published reports in which the adverse reaction has been described in detail
2. Did the adverse event appear after the suspected drug was administered?	+2	-1	0	2	Adverse reaction appeared soon after vaccination
3. Did the adverse reaction improve when the drug was discontinued, or a specific antagonist was administered?	+1	0	0	Score may be 0 to 1	Rapid improvement after immunomodulators administration for 3 of the patients. One of the patients did not need any drug remedy.
4. Did the adverse reaction reappear when the drug was readministered?	+2	-1	0	Score may be (-)1 to 0	3 patients had booster dose without any complaints
5. Are there alternative causes (other than the drug) that could on their own have caused the reaction?	-1	+2	0	0	
6. Did the reaction appeared when a placebo was given?	-1	+1	0	0	
7. Was the drug detected in the blood (or other body fluids) in concentrations known to be toxic?	+1	0	0	0	
8. Was the reaction more severe when the dose was increased, or less severe when dose decreased?	+1	0	0	0	
9. Did the patient have a similar reaction to the same or similar drugs in any previous exposure?	+1	0	0	0	
10. Was the adverse event confirmed by any objective evidence?	+1	0	0	Score may be 0 to 1	Biopsy conformation of AIH for 3 of the patients. One patient did not have liver biopsy.

Total score may be from **2 to 5** for each patient→**Possible/Probable adverse drug reaction**

**Case 6 by:** ***Barary et al***	Yes	No	Do not know	Score	Comment
1. Are there previous conclusive reports on this reaction?	+1	0	0	1	≥2 published reports in which the adverse reaction has been described in detail
2. Did the adverse event appear after the suspected drug was administered?	+2	-1	0	2	Adverse reaction appeared soon after vaccination
3. Did the adverse reaction improve when the drug was discontinued, or a specific antagonist was administered?	+1	0	0	0	No improvement after high-dose corticosteroids administration.
4. Did the adverse reaction reappear when the drug was readministered?	+2	-1	0	0	
5. Are there alternative causes (other than the drug) that could on their own have caused the reaction?	-1	+2	0	-1	The patient was treated with hepatotoxic agents for psychiatric disease
6. Did the reaction appeared when a placebo was given?	-1	+1	0	0	
7. Was the drug detected in the blood (or other body fluids) in concentrations known to be toxic?	+1	0	0	0	
8. Was the reaction more severe when the dose was increased, or less severe when dose decreased?	+1	0	0	0	
9. Did the patient have a similar reaction to the same or similar drugs in any previous exposure?	+1	0	0	0	
10. Was the adverse event confirmed by any objective evidence?	+1	0	0	0	Biopsy was not compatible with AIH

Total score: **2 →Possible adverse drug reaction**

Case 7 by: ***Mekritthikrai et al***	Yes	No	Do not know	Score	Comment
1. Are there previous conclusive reports on this reaction?	+1	0	0	1	≥2 published reports in which the adverse reaction has been described in detail
2. Did the adverse event appear after the suspected drug was administered?	+2	-1	0	2	Adverse reaction appeared soon after vaccination
3. Did the adverse reaction improve when the drug was discontinued, or a specific antagonist was administered?	+1	0	0	1	Rapid improvement after immunomodulator therapy
4. Did the adverse reaction reappear when the drug was readministered?	+2	-1	0	0	
5. Are there alternative causes (other than the drug) that could on their own have caused the reaction?	-1	+2	0	2	No known agent or condition that could provoke acute hepatitis
6. Did the reaction appeared when a placebo was given?	-1	+1	0	0	
7. Was the drug detected in the blood (or other body fluids) in concentrations known to be toxic?	+1	0	0	0	
8. Was the reaction more severe when the dose was increased, or less severe when dose decreased?	+1	0	0	0	
9. Did the patient have a similar reaction to the same or similar drugs in any previous exposure?	+1	0	0	0	
10. Was the adverse event confirmed by any objective evidence?	+1	0	0	1	Biopsy typical for AIH

Total score: **7** →**Probable adverse drug reaction**

Case 8 by: ***Brubaker et al***	Yes	No	Do not know	Score	Comment
1. Are there previous conclusive reports on this reaction?	+1	0	0	1	≥2 published reports in which the adverse reaction has been described in detail
2. Did the adverse event appear after the suspected drug was administered?	+2	-1	0	2	Adverse reaction appeared soon after vaccination
3. Did the adverse reaction improve when the drug was discontinued, or a specific antagonist was administered?	+1	0	0	0	
4. Did the adverse reaction reappear when the drug was readministered?	+2	-1	0	0	
5. Are there alternative causes (other than the drug) that could on their own have caused the reaction?	-1	+2	0	2	No known agent or condition that could flare of AIH
6. Did the reaction appeared when a placebo was given?	-1	+1	0	0	
7. Was the drug detected in the blood (or other body fluids) in concentrations known to be toxic?	+1	0	0	0	
8. Was the reaction more severe when the dose was increased, or less severe when dose decreased?	+1	0	0	0	
9. Did the patient have a similar reaction to the same or similar drugs in any previous exposure?	+1	0	0	0	
10. Was the adverse event confirmed by any objective evidence?	+1	0	0	1	Elevation of ASMA and hepatic enzymes. Biopsy confirmed AIH in the past.

Total score: **6** →**Probable adverse drug reaction**

Case 9 by: ***Shahrani et al***	Yes	No	Do not know	Score	Comment
1. Are there previous conclusive reports on this reaction?	+1	0	0	1	≥2 published reports in which the adverse reaction has been described in detail
2. Did the adverse event appear after the suspected drug was administered?	+2	-1	0	2	Adverse reaction appeared soon after vaccination
3. Did the adverse reaction improve when the drug was discontinued, or a specific antagonist was administered?	+1	0	0	1	Improvement with corticosteroids
4. Did the adverse reaction reappear when the drug was readministered?	+2	-1	0	0	
5. Are there alternative causes (other than the drug) that could on their own have caused the reaction?	-1	+2	0	2	No known agent or condition that could lead to AIH
6. Did the reaction appeared when a placebo was given?	-1	+1	0	0	
7. Was the drug detected in the blood (or other body fluids) in concentrations known to be toxic?	+1	0	0	0	
8. Was the reaction more severe when the dose was increased, or less severe when dose decreased?	+1	0	0	0	
9. Did the patient have a similar reaction to the same or similar drugs in any previous exposure?	+1	0	0	0	
10. Was the adverse event confirmed by any objective evidence?	+1	0	0	1	Biopsy compatible with AIH

Total score: **7** →**Probable adverse drug reaction**

Case 10 by: ***Shahrani et al***	Yes	No	Do not know	Score	Comment
1. Are there previous conclusive reports on this reaction?	+1	0	0	1	≥2 published reports in which the adverse reaction has been described in detail
2. Did the adverse event appear after the suspected drug was administered?	+2	-1	0	2	Adverse reaction appeared soon after vaccination
3. Did the adverse reaction improve when the drug was discontinued, or a specific antagonist was administered?	+1	0	0	1	Temporary improvement with corticosteroids
4. Did the adverse reaction reappear when the drug was readministered?	+2	-1	0	0	
5. Are there alternative causes (other than the drug) that could on their own have caused the reaction?	-1	+2	0	2	No known agent or condition that could lead to AIH
6. Did the reaction appeared when a placebo was given?	-1	+1	0	0	
7. Was the drug detected in the blood (or other body fluids) in concentrations known to be toxic?	+1	0	0	0	
8. Was the reaction more severe when the dose was increased, or less severe when dose decreased?	+1	0	0	0	
9. Did the patient have a similar reaction to the same or similar drugs in any previous exposure?	+1	0	0	0	
10. Was the adverse event confirmed by any objective evidence?	+1	0	0	1	Biopsy typical for AIH

Total score: **7** →**Probable adverse drug reaction**

**Case 11 by:** ***Shahrani et al***	Yes	No	Do not know	Score	Comment
1. Are there previous conclusive reports on this reaction?	+1	0	0	1	≥2 published reports in which the adverse reaction has been described in detail
2. Did the adverse event appear after the suspected drug was administered?	+2	-1	0	2	Adverse reaction appeared soon after vaccination
3. Did the adverse reaction improve when the drug was discontinued, or a specific antagonist was administered?	+1	0	0	1	Improvement with corticosteroids
4. Did the adverse reaction reappear when the drug was readministered?	+2	-1	0	0	
5. Are there alternative causes (other than the drug) that could on their own have caused the reaction?	-1	+2	0	2	No known agent or condition that could lead to AIH
6. Did the reaction appeared when a placebo was given?	-1	+1	0	0	
7. Was the drug detected in the blood (or other body fluids) in concentrations known to be toxic?	+1	0	0	0	
8. Was the reaction more severe when the dose was increased, or less severe when dose decreased?	+1	0	0	0	
9. Did the patient have a similar reaction to the same or similar drugs in any previous exposure?	+1	0	0	0	
10. Was the adverse event confirmed by any objective evidence?	+1	0	0	1	Biopsy compatible with AIH
Total score: **7** →**Probable adverse drug reaction**					

**Case 12 by:** ***Hasegawa et al***	Yes	No	Do not know	Score	Comment
1. Are there previous conclusive reports on this reaction?	+1	0	0	1	≥2 published reports in which the adverse reaction has been described in detail
2. Did the adverse event appear after the suspected drug was administered?	+2	-1	0	2	Adverse reaction appeared soon after vaccination
3. Did the adverse reaction improve when the drug was discontinued, or a specific antagonist was administered?	+1	0	0	1	Rapid improvement with immunomodulators
4. Did the adverse reaction reappear when the drug was readministered?	+2	-1	0	0	
5. Are there alternative causes (other than the drug) that could on their own have caused the reaction?	-1	+2	0	2	No known agent or condition that could lead to AIH
6. Did the reaction appeared when a placebo was given?	-1	+1	0	0	
7. Was the drug detected in the blood (or other body fluids) in concentrations known to be toxic?	+1	0	0	0	
8. Was the reaction more severe when the dose was increased, or less severe when dose decreased?	+1	0	0	0	
9. Did the patient have a similar reaction to the same or similar drugs in any previous exposure?	+1	0	0	0	
10. Was the adverse event confirmed by any objective evidence?	+1	0	0	1	Biopsy typical for AIH
Total score: **7** →**Probable adverse drug reaction**					

**Case 13 by:** ***Lasagna et al***	Yes	No	Do not know	Score	Comment
1. Are there previous conclusive reports on this reaction?	+1	0	0	1	≥2 published reports in which the adverse reaction has been described in detail
2. Did the adverse event appear after the suspected drug was administered?	+2	-1	0	2	Adverse reaction appeared soon after vaccination
3. Did the adverse reaction improve when the drug was discontinued, or a specific antagonist was administered?	+1	0	0	1	Rapid improvement with immunomodulators
4. Did the adverse reaction reappear when the drug was readministered?	+2	-1	0	0	
5. Are there alternative causes (other than the drug) that could on their own have caused the reaction?	-1	+2	0	-1	Cancer patient on immunotherapy
6. Did the reaction appeared when a placebo was given?	-1	+1	0	0	
7. Was the drug detected in the blood (or other body fluids) in concentrations known to be toxic?	+1	0	0	0	
8. Was the reaction more severe when the dose was increased, or less severe when dose decreased?	+1	0	0	0	
9. Did the patient have a similar reaction to the same or similar drugs in any previous exposure?	+1	0	0	0	
10. Was the adverse event confirmed by any objective evidence?	+1	0	0	0	Biopsy non-consistent with AIH
Total score: **3** →**Possible adverse drug reaction**					

**Case 14 by:** ***Pinazo-Bandera et al***	Yes	No	Do not know	Score	Comment
1. Are there previous conclusive reports on this reaction?	+1	0	0	1	≥2 published reports in which the adverse reaction has been described in detail
2. Did the adverse event appear after the suspected drug was administered?	+2	-1	0	2	Adverse reaction appeared soon after vaccination
3. Did the adverse reaction improve when the drug was discontinued, or a specific antagonist was administered?	+1	0	0	1	Improvement with immunomodulators
4. Did the adverse reaction reappear when the drug was readministered?	+2	-1	0	0	
5. Are there alternative causes (other than the drug) that could on their own have caused the reaction?	-1	+2	0	2	No known agent or condition that could lead to AIH
6. Did the reaction appeared when a placebo was given?	-1	+1	0	0	
7. Was the drug detected in the blood (or other body fluids) in concentrations known to be toxic?	+1	0	0	0	
8. Was the reaction more severe when the dose was increased, or less severe when dose decreased?	+1	0	0	0	
9. Did the patient have a similar reaction to the same or similar drugs in any previous exposure?	+1	0	0	0	
10. Was the adverse event confirmed by any objective evidence?	+1	0	0	1	Biopsy consistent with AIH
Total score: **7** →**Probable adverse drug reaction**					

Case 15 by: ***Pinazo-Bandera et al***	Yes	No	Do not know	Score	Comment
1. Are there previous conclusive reports on this reaction?	+1	0	0	1	≥2 published reports in which the adverse reaction has been described in detail
2. Did the adverse event appear after the suspected drug was administered?	+2	-1	0	2	Adverse reaction appeared soon after vaccination
3. Did the adverse reaction improve when the drug was discontinued, or a specific antagonist was administered?	+1	0	0	1	Improvement with immunomodulators
4. Did the adverse reaction reappear when the drug was readministered?	+2	-1	0	0	
5. Are there alternative causes (other than the drug) that could on their own have caused the reaction?	-1	+2	0	2	No known agent or condition that could lead to AIH
6. Did the reaction appeared when a placebo was given?	-1	+1	0	0	
7. Was the drug detected in the blood (or other body fluids) in concentrations known to be toxic?	+1	0	0	0	
8. Was the reaction more severe when the dose was increased, or less severe when dose decreased?	+1	0	0	0	
9. Did the patient have a similar reaction to the same or similar drugs in any previous exposure?	+1	0	0	0	
10. Was the adverse event confirmed by any objective evidence?	+1	0	0	1	Biopsy consistent with AIH

Total score: **7** →**Probable adverse drug reaction**

Case 16 by: ***Boettler et al***	Yes	No	Do not know	Score	Comment
1. Are there previous conclusive reports on this reaction?	+1	0	0	1	≥2 published reports in which the adverse reaction has been described in detail
2. Did the adverse event appear after the suspected drug was administered?	+2	-1	0	2	Adverse reaction appeared soon after vaccination
3. Did the adverse reaction improve when the drug was discontinued, or a specific antagonist was administered?	+1	0	0	1	Improvement with corticosteroids
4. Did the adverse reaction reappear when the drug was readministered?	+2	-1	0	2	
5. Are there alternative causes (other than the drug) that could on their own have caused the reaction?	-1	+2	0	2	No known agent or condition that could lead to AIH
6. Did the reaction appeared when a placebo was given?	-1	+1	0	0	
7. Was the drug detected in the blood (or other body fluids) in concentrations known to be toxic?	+1	0	0	0	
8. Was the reaction more severe when the dose was increased, or less severe when dose decreased?	+1	0	0	0	
9. Did the patient have a similar reaction to the same or similar drugs in any previous exposure?	+1	0	0	0	
10. Was the adverse event confirmed by any objective evidence?	+1	0	0	1	Biopsy consistent with AIH

Total score: **9** →**Definite adverse drug reaction**

Case 17 by: ***Kang et al***	Yes	No	Do not know	Score	Comment
1. Are there previous conclusive reports on this reaction?	+1	0	0	1	≥2 published reports in which the adverse reaction has been described in detail
2. Did the adverse event appear after the suspected drug was administered?	+2	-1	0	2	Adverse reaction appeared soon after vaccination
3. Did the adverse reaction improve when the drug was discontinued, or a specific antagonist was administered?	+1	0	0	1	Improvement with corticosteroids
4. Did the adverse reaction reappear when the drug was readministered?	+2	-1	0	0	
5. Are there alternative causes (other than the drug) that could on their own have caused the reaction?	-1	+2	0	2	No known agent or condition that could lead to AIH
6. Did the reaction appeared when a placebo was given?	-1	+1	0	0	
7. Was the drug detected in the blood (or other body fluids) in concentrations known to be toxic?	+1	0	0	0	
8. Was the reaction more severe when the dose was increased, or less severe when dose decreased?	+1	0	0	0	
9. Did the patient have a similar reaction to the same or similar drugs in any previous exposure?	+1	0	0	0	
10. Was the adverse event confirmed by any objective evidence?	+1	0	0	1	Biopsy typical for AIH

Total score: **7** →**Probable adverse drug reaction**

Case 18 by: ***Ghorbani et al***	Yes	No	Do not know	Score	Comment
1. Are there previous conclusive reports on this reaction?	+1	0	0	1	≥2 published reports in which the adverse reaction has been described in detail
2. Did the adverse event appear after the suspected drug was administered?	+2	-1	0	2	Adverse reaction appeared soon after vaccination
3. Did the adverse reaction improve when the drug was discontinued, or a specific antagonist was administered?	+1	0	0	0	
4. Did the adverse reaction reappear when the drug was readministered?	+2	-1	0	0	
5. Are there alternative causes (other than the drug) that could on their own have caused the reaction?	-1	+2	0	2	No known agent or condition that could lead to AIH
6. Did the reaction appeared when a placebo was given?	-1	+1	0	0	
7. Was the drug detected in the blood (or other body fluids) in concentrations known to be toxic?	+1	0	0	0	
8. Was the reaction more severe when the dose was increased, or less severe when dose decreased?	+1	0	0	0	
9. Did the patient have a similar reaction to the same or similar drugs in any previous exposure?	+1	0	0	0	
10. Was the adverse event confirmed by any objective evidence?	+1	0	0	1	Biopsy compatible with AIH

Total score: **6** →**Probable adverse drug reaction**

**Case 19 by:** ***Fimiano et al***	Yes	No	Do not know	Score	Comment
1. Are there previous conclusive reports on this reaction?	+1	0	0	1	≥2 published reports in which the adverse reaction has been described in detail
2. Did the adverse event appear after the suspected drug was administered?	+2	-1	0	2	Adverse reaction appeared soon after vaccination
3. Did the adverse reaction improve when the drug was discontinued, or a specific antagonist was administered?	+1	0	0	1	Improvement with corticosteroids
4. Did the adverse reaction reappear when the drug was readministered?	+2	-1	0	0	
5. Are there alternative causes (other than the drug) that could on their own have caused the reaction?	-1	+2	0	2	No known agent or condition that could lead to AIH
6. Did the reaction appeared when a placebo was given?	-1	+1	0	0	
7. Was the drug detected in the blood (or other body fluids) in concentrations known to be toxic?	+1	0	0	0	
8. Was the reaction more severe when the dose was increased, or less severe when dose decreased?	+1	0	0	0	
9. Did the patient have a similar reaction to the same or similar drugs in any previous exposure?	+1	0	0	0	
10. Was the adverse event confirmed by any objective evidence?	+1	0	0	1	Biopsy compatible with AIH

Total score: **7** → **Probable adverse drug reaction**

**Case 20 by:** ***Camacho-Dominguez et al***	Yes	No	Do not know	Score	Comment
1. Are there previous conclusive reports on this reaction?	+1	0	0	1	≥2 published reports in which the adverse reaction has been described in detail
2. Did the adverse event appear after the suspected drug was administered?	+2	-1	0	2	Adverse reaction appeared soon after vaccination
3. Did the adverse reaction improve when the drug was discontinued, or a specific antagonist was administered?	+1	0	0	1	Improvement with corticosteroids
4. Did the adverse reaction reappear when the drug was readministered?	+2	-1	0	0	
5. Are there alternative causes (other than the drug) that could on their own have caused the reaction?	-1	+2	0	2	No known agent or condition that could lead to AIH
6. Did the reaction appeared when a placebo was given?	-1	+1	0	0	
7. Was the drug detected in the blood (or other body fluids) in concentrations known to be toxic?	+1	0	0	0	
8. Was the reaction more severe when the dose was increased, or less severe when dose decreased?	+1	0	0	0	
9. Did the patient have a similar reaction to the same or similar drugs in any previous exposure?	+1	0	0	0	
10. Was the adverse event confirmed by any objective evidence?	+1	0	0	1	Biopsy compatible with AIH

Total score: **7** → **Probable adverse drug reaction**

**Cases 21,22 by:** ***Erard et al***	Yes	No	Do not know	Score	Comment
1. Are there previous conclusive reports on this reaction?	+1	0	0	1	≥2 published reports in which the adverse reaction has been described in detail
2. Did the adverse event appear after the suspected drug was administered?	+2	-1	0	2	Adverse reaction appeared soon after vaccination
3. Did the adverse reaction improve when the drug was discontinued, or a specific antagonist was administered?	+1	0	0	1	Improvement with corticosteroids
4. Did the adverse reaction reappear when the drug was readministered?	+2	-1	0	0	
5. Are there alternative causes (other than the drug) that could on their own have caused the reaction?	-1	+2	0	2	No known agent or condition that could lead to AIH
6. Did the reaction appeared when a placebo was given?	-1	+1	0	0	
7. Was the drug detected in the blood (or other body fluids) in concentrations known to be toxic?	+1	0	0	0	
8. Was the reaction more severe when the dose was increased, or less severe when dose decreased?	+1	0	0	0	
9. Did the patient have a similar reaction to the same or similar drugs in any previous exposure?	+1	0	0	0	
10. Was the adverse event confirmed by any objective evidence?	+1	0	0	1	Biopsy compatible with AIH

Total score: **7** → **Probable adverse drug reaction**

**Case 23 by:** ***Erard et al***	Yes	No	Do not know	Score	Comment
1. Are there previous conclusive reports on this reaction?	+1	0	0	1	≥2 published reports in which the adverse reaction has been described in detail
2. Did the adverse event appear after the suspected drug was administered?	+2	-1	0	2	Adverse reaction appeared soon after vaccination
3. Did the adverse reaction improve when the drug was discontinued, or a specific antagonist was administered?	+1	0	0	0	No improvement with immunomodulators
4. Did the adverse reaction reappear when the drug was readministered?	+2	-1	0	0	
5. Are there alternative causes (other than the drug) that could on their own have caused the reaction?	-1	+2	0	2	No known agent or condition that could lead to AIH
6. Did the reaction appeared when a placebo was given?	-1	+1	0	0	
7. Was the drug detected in the blood (or other body fluids) in concentrations known to be toxic?	+1	0	0	0	
8. Was the reaction more severe when the dose was increased, or less severe when dose decreased?	+1	0	0	0	
9. Did the patient have a similar reaction to the same or similar drugs in any previous exposure?	+1	0	0	0	
10. Was the adverse event confirmed by any objective evidence?	+1	0	0	1	Biopsy compatible with AIH

Total score: **6** → **Probable adverse drug reaction**

**Cases 24, 25, 26 by:** ***Suzuki et al***	Yes	No	Do not know	Score	Comment
1. Are there previous conclusive reports on this reaction?	+1	0	0	1	≥2 published reports in which the adverse reaction has been described in detail
2. Did the adverse event appear after the suspected drug was administered?	+2	-1	0	2	Adverse reaction appeared soon after vaccination
3. Did the adverse reaction improve when the drug was discontinued, or a specific antagonist was administered?	+1	0	0	1	Resolution with corticosteroids
4. Did the adverse reaction reappear when the drug was readministered?	+2	-1	0	0	
5. Are there alternative causes (other than the drug) that could on their own have caused the reaction?	-1	+2	0	2	No known agent or condition that could lead to AIH
6. Did the reaction appeared when a placebo was given?	-1	+1	0	0	
7. Was the drug detected in the blood (or other body fluids) in concentrations known to be toxic?	+1	0	0	0	
8. Was the reaction more severe when the dose was increased, or less severe when dose decreased?	+1	0	0	0	
9. Did the patient have a similar reaction to the same or similar drugs in any previous exposure?	+1	0	0	0	
10. Was the adverse event confirmed by any objective evidence?	+1	0	0	1	Biopsy compatible with AIH

Total score: **7** → **Probable adverse drug reaction**

**Case 27 by:** ***Cao et al***	Yes	No	Do not know	Score	Comment
1. Are there previous conclusive reports on this reaction?	+1	0	0	1	≥2 published reports in which the adverse reaction has been described in detail
2. Did the adverse event appear after the suspected drug was administered?	+2	-1	0	2	Adverse reaction appeared soon after vaccination
3. Did the adverse reaction improve when the drug was discontinued, or a specific antagonist was administered?	+1	0	0	1	Resolution with corticosteroids
4. Did the adverse reaction reappear when the drug was readministered?	+2	-1	0	0	
5. Are there alternative causes (other than the drug) that could on their own have caused the reaction?	-1	+2	0	2	No known agent or condition that could lead to AIH
6. Did the reaction appeared when a placebo was given?	-1	+1	0	0	
7. Was the drug detected in the blood (or other body fluids) in concentrations known to be toxic?	+1	0	0	0	
8. Was the reaction more severe when the dose was increased, or less severe when dose decreased?	+1	0	0	0	
9. Did the patient have a similar reaction to the same or similar drugs in any previous exposure?	+1	0	0	0	
10. Was the adverse event confirmed by any objective evidence?	+1	0	0	1	Biopsy compatible with AIH

Total score: **7** → **Probable adverse drug reaction**

**Case 28 by:** ***Avci et al***	Yes	No	Do not know	Score	Comment
1. Are there previous conclusive reports on this reaction?	+1	0	0	1	≥2 published reports in which the adverse reaction has been described in detail
2. Did the adverse event appear after the suspected drug was administered?	+2	-1	0	2	Adverse reaction appeared soon after vaccination
3. Did the adverse reaction improve when the drug was discontinued, or a specific antagonist was administered?	+1	0	0	1	Resolution with corticosteroids
4. Did the adverse reaction reappear when the drug was readministered?	+2	-1	0	0	
5. Are there alternative causes (other than the drug) that could on their own have caused the reaction?	-1	+2	0	2	No known agent or condition that could lead to AIH
6. Did the reaction appeared when a placebo was given?	-1	+1	0	0	
7. Was the drug detected in the blood (or other body fluids) in concentrations known to be toxic?	+1	0	0	0	
8. Was the reaction more severe when the dose was increased, or less severe when dose decreased?	+1	0	0	0	
9. Did the patient have a similar reaction to the same or similar drugs in any previous exposure?	+1	0	0	0	
10. Was the adverse event confirmed by any objective evidence?	+1	0	0	1	Biopsy compatible with AIH

Total score: **7** → **Probable adverse drug reaction**

**Case 29 by:** ***Garrido et al***	Yes	No	Do not know	Score	Comment
1. Are there previous conclusive reports on this reaction?	+1	0	0	1	≥2 published reports in which the adverse reaction has been described in detail
2. Did the adverse event appear after the suspected drug was administered?	+2	-1	0	2	Adverse reaction appeared soon after vaccination
3. Did the adverse reaction improve when the drug was discontinued, or a specific antagonist was administered?	+1	0	0	1	Resolution with corticosteroids
4. Did the adverse reaction reappear when the drug was readministered?	+2	-1	0	0	
5. Are there alternative causes (other than the drug) that could on their own have caused the reaction?	-1	+2	0	2	No known agent or condition that could lead to AIH
6. Did the reaction appeared when a placebo was given?	-1	+1	0	0	
7. Was the drug detected in the blood (or other body fluids) in concentrations known to be toxic?	+1	0	0	0	
8. Was the reaction more severe when the dose was increased, or less severe when dose decreased?	+1	0	0	0	
9. Did the patient have a similar reaction to the same or similar drugs in any previous exposure?	+1	0	0	0	
10. Was the adverse event confirmed by any objective evidence?	+1	0	0	1	Biopsy compatible with AIH

Total score: **7** → **Probable adverse drug reaction**

**Case 30 by:** ***Palla et al***	Yes	No	Do not know	Score	Comment
1. Are there previous conclusive reports on this reaction?	+1	0	0	1	≥2 published reports in which the adverse reaction has been described in detail
2. Did the adverse event appear after the suspected drug was administered?	+2	-1	0	2	Adverse reaction appeared soon after vaccination
3. Did the adverse reaction improve when the drug was discontinued, or a specific antagonist was administered?	+1	0	0	1	Resolution with corticosteroids
4. Did the adverse reaction reappear when the drug was readministered?	+2	-1	0	0	
5. Are there alternative causes (other than the drug) that could on their own have caused the reaction?	-1	+2	0	2	No known agent or condition that could lead to AIH
6. Did the reaction appeared when a placebo was given?	-1	+1	0	0	
7. Was the drug detected in the blood (or other body fluids) in concentrations known to be toxic?	+1	0	0	0	
8. Was the reaction more severe when the dose was increased, or less severe when dose decreased?	+1	0	0	0	
9. Did the patient have a similar reaction to the same or similar drugs in any previous exposure?	+1	0	0	0	
10. Was the adverse event confirmed by any objective evidence?	+1	0	0	1	Biopsy compatible with AIH
Total score: 7 → **Probable adverse drug reaction**					

**Case 31 by:** ***Zhou et al***	Yes	No	Do not know	Score	Comment
1. Are there previous conclusive reports on this reaction?	+1	0	0	1	≥2 published reports in which the adverse reaction has been described in detail
2. Did the adverse event appear after the suspected drug was administered?	+2	-1	0	2	Adverse reaction appeared soon after vaccination
3. Did the adverse reaction improve when the drug was discontinued, or a specific antagonist was administered?	+1	0	0	1	Resolution with corticosteroids
4. Did the adverse reaction reappear when the drug was readministered?	+2	-1	0	0	
5. Are there alternative causes (other than the drug) that could on their own have caused the reaction?	-1	+2	0	2	Patient had a diagnosis of primary sclerosing cholangitis (PSC). However, she was on remission under continuous monitoring and flare of PSC is considered unlikely.
6. Did the reaction appeared when a placebo was given?	-1	+1	0	0	
7. Was the drug detected in the blood (or other body fluids) in concentrations known to be toxic?	+1	0	0	0	
8. Was the reaction more severe when the dose was increased, or less severe when dose decreased?	+1	0	0	0	
9. Did the patient have a similar reaction to the same or similar drugs in any previous exposure?	+1	0	0	0	
10. Was the adverse event confirmed by any objective evidence?	+1	0	0	1	Biopsy compatible with AIH
Total score: 7 → **Probable adverse drug reaction**					

**Case 32 by:** ***Ghielmetti et al***	Yes	No	Do not know	Score	Comment
1. Are there previous conclusive reports on this reaction?	+1	0	0	1	≥2 published reports in which the adverse reaction has been described in detail
2. Did the adverse event appear after the suspected drug was administered?	+2	-1	0	2	Adverse reaction appeared soon after vaccination
3. Did the adverse reaction improve when the drug was discontinued, or a specific antagonist was administered?	+1	0	0	1	Resolution with corticosteroids
4. Did the adverse reaction reappear when the drug was readministered?	+2	-1	0	0	
5. Are there alternative causes (other than the drug) that could on their own have caused the reaction?	-1	+2	0	2	No known agent or condition that could lead to AIH
6. Did the reaction appeared when a placebo was given?	-1	+1	0	0	
7. Was the drug detected in the blood (or other body fluids) in concentrations known to be toxic?	+1	0	0	0	
8. Was the reaction more severe when the dose was increased, or less severe when dose decreased?	+1	0	0	0	
9. Did the patient have a similar reaction to the same or similar drugs in any previous exposure?	+1	0	0	0	
10. Was the adverse event confirmed by any objective evidence?	+1	0	0	1	Biopsy compatible with AIH
Total score: 7 → **Probable adverse drug reaction**					

**Case 33 by:** ***McShane et al***	Yes	No	Do not know	Score	Comment
1. Are there previous conclusive reports on this reaction?	+1	0	0	1	≥2 published reports in which the adverse reaction has been described in detail
2. Did the adverse event appear after the suspected drug was administered?	+2	-1	0	2	Adverse reaction appeared soon after vaccination
3. Did the adverse reaction improve when the drug was discontinued, or a specific antagonist was administered?	+1	0	0	1	Resolution with corticosteroids
4. Did the adverse reaction reappear when the drug was readministered?	+2	-1	0	0	
5. Are there alternative causes (other than the drug) that could on their own have caused the reaction?	-1	+2	0	2	No known agent or condition that could lead to AIH
6. Did the reaction appeared when a placebo was given?	-1	+1	0	0	
7. Was the drug detected in the blood (or other body fluids) in concentrations known to be toxic?	+1	0	0	0	
8. Was the reaction more severe when the dose was increased, or less severe when dose decreased?	+1	0	0	0	
9. Did the patient have a similar reaction to the same or similar drugs in any previous exposure?	+1	0	0	0	
10. Was the adverse event confirmed by any objective evidence?	+1	0	0	1	Biopsy compatible with AIH
Total score: 7 → **Probable adverse drug reaction**					

**Case 34 by:** ***Clayton-Chubb et al***	Yes	No	Do not know	Score	Comment
1. Are there previous conclusive reports on this reaction?	+1	0	0	1	≥2 published reports in which the adverse reaction has been described in detail
2. Did the adverse event appear after the suspected drug was administered?	+2	-1	0	2	Adverse reaction appeared soon after vaccination
3. Did the adverse reaction improve when the drug was discontinued, or a specific antagonist was administered?	+1	0	0	1	Resolution with corticosteroids
4. Did the adverse reaction reappear when the drug was readministered?	+2	-1	0	0	
5. Are there alternative causes (other than the drug) that could on their own have caused the reaction?	-1	+2	0	2	No known agent or condition that could lead to AIH
6. Did the reaction appeared when a placebo was given?	-1	+1	0	0	
7. Was the drug detected in the blood (or other body fluids) in concentrations known to be toxic?	+1	0	0	0	
8. Was the reaction more severe when the dose was increased, or less severe when dose decreased?	+1	0	0	0	
9. Did the patient have a similar reaction to the same or similar drugs in any previous exposure?	+1	0	0	0	
10. Was the adverse event confirmed by any objective evidence?	+1	0	0	1	Biopsy compatible with AIH

Total score: **7** → **Probable adverse drug reaction**

**Case 35 by:** ***Tan et al***	Yes	No	Do not know	Score	Comment
1. Are there previous conclusive reports on this reaction?	+1	0	0	1	≥2 published reports in which the adverse reaction has been described in detail
2. Did the adverse event appear after the suspected drug was administered?	+2	-1	0	2	Adverse reaction appeared soon after vaccination
3. Did the adverse reaction improve when the drug was discontinued, or a specific antagonist was administered?	+1	0	0	1	Resolution with corticosteroids
4. Did the adverse reaction reappear when the drug was readministered?	+2	-1	0	0	
5. Are there alternative causes (other than the drug) that could on their own have caused the reaction?	-1	+2	0	2	No known agent or condition that could lead to AIH
6. Did the reaction appeared when a placebo was given?	-1	+1	0	0	
7. Was the drug detected in the blood (or other body fluids) in concentrations known to be toxic?	+1	0	0	0	
8. Was the reaction more severe when the dose was increased, or less severe when dose decreased?	+1	0	0	0	
9. Did the patient have a similar reaction to the same or similar drugs in any previous exposure?	+1	0	0	0	
10. Was the adverse event confirmed by any objective evidence?	+1	0	0	1	Biopsy compatible with AIH

Total score: **7** → **Probable adverse drug reaction**

**Case 36 by:** ***Rocco et al***	Yes	No	Do not know	Score	Comment
1. Are there previous conclusive reports on this reaction?	+1	0	0	1	≥2 published reports in which the adverse reaction has been described in detail
2. Did the adverse event appear after the suspected drug was administered?	+2	-1	0	2	Adverse reaction appeared soon after vaccination
3. Did the adverse reaction improve when the drug was discontinued, or a specific antagonist was administered?	+1	0	0	1	Improvement with corticosteroids
4. Did the adverse reaction reappear when the drug was readministered?	+2	-1	0	0	
5. Are there alternative causes (other than the drug) that could on their own have caused the reaction?	-1	+2	0	2	No known agent or condition that could lead to AIH
6. Did the reaction appeared when a placebo was given?	-1	+1	0	0	
7. Was the drug detected in the blood (or other body fluids) in concentrations known to be toxic?	+1	0	0	0	
8. Was the reaction more severe when the dose was increased, or less severe when dose decreased?	+1	0	0	0	
9. Did the patient have a similar reaction to the same or similar drugs in any previous exposure?	+1	0	0	0	
10. Was the adverse event confirmed by any objective evidence?	+1	0	0	1	Biopsy compatible with AIH

Total score: **7** → **Probable adverse drug reaction**

**Case 37 by:** ***Bril et al***	Yes	No	Do not know	Score	Comment
1. Are there previous conclusive reports on this reaction?	+1	0	0	1	≥2 published reports in which the adverse reaction has been described in detail
2. Did the adverse event appear after the suspected drug was administered?	+2	-1	0	2	Adverse reaction appeared soon after vaccination
3. Did the adverse reaction improve when the drug was discontinued, or a specific antagonist was administered?	+1	0	0	1	Improvement with corticosteroids
4. Did the adverse reaction reappear when the drug was readministered?	+2	-1	0	0	
5. Are there alternative causes (other than the drug) that could on their own have caused the reaction?	-1	+2	0	2	No known agent or condition that could lead to AIH
6. Did the reaction appeared when a placebo was given?	-1	+1	0	0	
7. Was the drug detected in the blood (or other body fluids) in concentrations known to be toxic?	+1	0	0	0	
8. Was the reaction more severe when the dose was increased, or less severe when dose decreased?	+1	0	0	0	
9. Did the patient have a similar reaction to the same or similar drugs in any previous exposure?	+1	0	0	0	
10. Was the adverse event confirmed by any objective evidence?	+1	0	0	1	Biopsy compatible with AIH

Total score: **7** → **Probable adverse drug reaction**

Case 38 by: *Goulas et al*	Yes	No	Do not know	Score	Comment
1. Are there previous conclusive reports on this reaction?	+1	0	0	1	≥2 published reports in which the adverse reaction has been described in detail
2. Did the adverse event appear after the suspected drug was administered?	+2	-1	0	2	Adverse reaction appeared soon after vaccination
3. Did the adverse reaction improve when the drug was discontinued, or a specific antagonist was administered?	+1	0	0	1	Improvement with corticosteroids
4. Did the adverse reaction reappear when the drug was readministered?	+2	-1	0	0	
5. Are there alternative causes (other than the drug) that could on their own have caused the reaction?	-1	+2	0	2	No known agent or condition that could lead to AIH
6. Did the reaction appeared when a placebo was given?	-1	+1	0	0	
7. Was the drug detected in the blood (or other body fluids) in concentrations known to be toxic?	+1	0	0	0	
8. Was the reaction more severe when the dose was increased, or less severe when dose decreased?	+1	0	0	0	
9. Did the patient have a similar reaction to the same or similar drugs in any previous exposure?	+1	0	0	0	
10. Was the adverse event confirmed by any objective evidence?	+1	0	0	1	Biopsy compatible with AIH
Total score: **7** → **Probable adverse drug reaction**					

Case 39 by: *Lodato et al*	Yes	No	Do not know	Score	Comment
1. Are there previous conclusive reports on this reaction?	+1	0	0	1	≥2 published reports in which the adverse reaction has been described in detail
2. Did the adverse event appear after the suspected drug was administered?	+2	-1	0	2	Adverse reaction appeared soon after vaccination
3. Did the adverse reaction improve when the drug was discontinued, or a specific antagonist was administered?	+1	0	0	1	Resolution with corticosteroids
4. Did the adverse reaction reappear when the drug was readministered?	+2	-1	0	0	
5. Are there alternative causes (other than the drug) that could on their own have caused the reaction?	-1	+2	0	2	No known agent or condition that could lead to AIH
6. Did the reaction appeared when a placebo was given?	-1	+1	0	0	
7. Was the drug detected in the blood (or other body fluids) in concentrations known to be toxic?	+1	0	0	0	
8. Was the reaction more severe when the dose was increased, or less severe when dose decreased?	+1	0	0	0	
9. Did the patient have a similar reaction to the same or similar drugs in any previous exposure?	+1	0	0	0	
10. Was the adverse event confirmed by any objective evidence?	+1	0	0	1	Biopsy compatible with AIH
Total score: 7 → **Probable adverse drug reaction**					

Case 40 by: *Londono et al*	Yes	No	Do not know	Score	Comment
1. Are there previous conclusive reports on this reaction?	+1	0	0	1	≥2 published reports in which the adverse reaction has been described in detail
2. Did the adverse event appear after the suspected drug was administered?	+2	-1	0	2	Adverse reaction appeared soon after vaccination
3. Did the adverse reaction improve when the drug was discontinued, or a specific antagonist was administered?	+1	0	0	1	Resolution with corticosteroids
4. Did the adverse reaction reappear when the drug was readministered?	+2	-1	0	2	Precipitation of symptoms after second vaccination
5. Are there alternative causes (other than the drug) that could on their own have caused the reaction?	-1	+2	0	2	No known agent or condition that could lead to AIH
6. Did the reaction appeared when a placebo was given?	-1	+1	0	0	
7. Was the drug detected in the blood (or other body fluids) in concentrations known to be toxic?	+1	0	0	0	
8. Was the reaction more severe when the dose was increased, or less severe when dose decreased?	+1	0	0	0	
9. Did the patient have a similar reaction to the same or similar drugs in any previous exposure?	+1	0	0	0	
10. Was the adverse event confirmed by any objective evidence?	+1	0	0	1	Biopsy compatible with AIH
Total score: **9** → **Definite adverse drug reaction**					

Case 41 by: *Rela et al*	Yes	No	Do not know	Score	Comment
1. Are there previous conclusive reports on this reaction?	+1	0	0	1	≥2 published reports in which the adverse reaction has been described in detail
2. Did the adverse event appear after the suspected drug was administered?	+2	-1	0	2	Adverse reaction appeared soon after vaccination
3. Did the adverse reaction improve when the drug was discontinued, or a specific antagonist was administered?	+1	0	0	1	Resolution with corticosteroids
4. Did the adverse reaction reappear when the drug was readministered?	+2	-1	0	0	
5. Are there alternative causes (other than the drug) that could on their own have caused the reaction?	-1	+2	0	2	No known agent or condition that could lead to AIH
6. Did the reaction appeared when a placebo was given?	-1	+1	0	0	
7. Was the drug detected in the blood (or other body fluids) in concentrations known to be toxic?	+1	0	0	0	
8. Was the reaction more severe when the dose was increased, or less severe when dose decreased?	+1	0	0	0	
9. Did the patient have a similar reaction to the same or similar drugs in any previous exposure?	+1	0	0	0	
10. Was the adverse event confirmed by any objective evidence?	+1	0	0	1	Biopsy compatible with AIH
Total score: 7 → **Probable adverse drug reaction**					

**Case 42 by:** ***Rela et al***	Yes	No	Do not know	Score	Comment
1. Are there previous conclusive reports on this reaction?	+1	0	0	1	≥2 published reports in which the adverse reaction has been described in detail
2. Did the adverse event appear after the suspected drug was administered?	+2	-1	0	2	Adverse reaction appeared soon after vaccination
3. Did the adverse reaction improve when the drug was discontinued, or a specific antagonist was administered?	+1	0	0	0	Poor response to corticosteroids
4. Did the adverse reaction reappear when the drug was readministered?	+2	-1	0	0	
5. Are there alternative causes (other than the drug) that could on their own have caused the reaction?	-1	+2	0	-1	Medical history of jaundice episodes
6. Did the reaction appeared when a placebo was given?	-1	+1	0	0	
7. Was the drug detected in the blood (or other body fluids) in concentrations known to be toxic?	+1	0	0	0	
8. Was the reaction more severe when the dose was increased, or less severe when dose decreased?	+1	0	0	0	
9. Did the patient have a similar reaction to the same or similar drugs in any previous exposure?	+1	0	0	0	
10. Was the adverse event confirmed by any objective evidence?	+1	0	0	1	Biopsy compatible with AIH

Total score: **3** → **Possible adverse drug reaction**

**Case 43 by:** ***Zin Tun et al***	Yes	No	Do not know	Score	Comment
1. Are there previous conclusive reports on this reaction?	+1	0	0	1	≥2 published reports in which the adverse reaction has been described in detail
2. Did the adverse event appear after the suspected drug was administered?	+2	-1	0	2	Adverse reaction appeared soon after vaccination
3. Did the adverse reaction improve when the drug was discontinued, or a specific antagonist was administered?	+1	0	0	1	Resolution with corticosteroids
4. Did the adverse reaction reappear when the drug was readministered?	+2	-1	0	2	
5. Are there alternative causes (other than the drug) that could on their own have caused the reaction?	-1	+2	0	2	No known agent or condition that could lead to AIH
6. Did the reaction appeared when a placebo was given?	-1	+1	0	0	
7. Was the drug detected in the blood (or other body fluids) in concentrations known to be toxic?	+1	0	0	0	
8. Was the reaction more severe when the dose was increased, or less severe when dose decreased?	+1	0	0	0	
9. Did the patient have a similar reaction to the same or similar drugs in any previous exposure?	+1	0	0	0	
10. Was the adverse event confirmed by any objective evidence?	+1	0	0	1	Biopsy compatible with AIH

Total score: **9** → **Probable adverse drug reaction**

**Case 44 by:** ***Vuille-Lessard et al***	Yes	No	Do not know	Score	Comment
1. Are there previous conclusive reports on this reaction?	+1	0	0	1	≥2 published reports in which the adverse reaction has been described in detail
2. Did the adverse event appear after the suspected drug was administered?	+2	-1	0	2	Adverse reaction appeared soon after vaccination
3. Did the adverse reaction improve when the drug was discontinued, or a specific antagonist was administered?	+1	0	0	1	Resolution with immunomodulators
4. Did the adverse reaction reappear when the drug was readministered?	+2	-1	0	0	
5. Are there alternative causes (other than the drug) that could on their own have caused the reaction?	-1	+2	0	2	No known agent or condition that could lead to AIH
6. Did the reaction appeared when a placebo was given?	-1	+1	0	0	
7. Was the drug detected in the blood (or other body fluids) in concentrations known to be toxic?	+1	0	0	0	
8. Was the reaction more severe when the dose was increased, or less severe when dose decreased?	+1	0	0	0	
9. Did the patient have a similar reaction to the same or similar drugs in any previous exposure?	+1	0	0	0	
10. Was the adverse event confirmed by any objective evidence?	+1	0	0	1	Biopsy compatible with AIH

Total score: **7** → **Probable adverse drug reaction**

**Case 45 by:** ***Mathew et al***	Yes	No	Do not know	Score	Comment
1. Are there previous conclusive reports on this reaction?	+1	0	0	1	≥2 published reports in which the adverse reaction has been described in detail
2. Did the adverse event appear after the suspected drug was administered?	+2	-1	0	2	Adverse reaction appeared soon after vaccination
3. Did the adverse reaction improve when the drug was discontinued, or a specific antagonist was administered?	+1	0	0	1	Resolution with immunomodulators
4. Did the adverse reaction reappear when the drug was readministered?	+2	-1	0	0	
5. Are there alternative causes (other than the drug) that could on their own have caused the reaction?	-1	+2	0	-1	Chronic use of analgetics. Possible drug toxicity is described also in liver histopathological findings.
6. Did the reaction appeared when a placebo was given?	-1	+1	0	0	
7. Was the drug detected in the blood (or other body fluids) in concentrations known to be toxic?	+1	0	0	0	
8. Was the reaction more severe when the dose was increased, or less severe when dose decreased?	+1	0	0	0	
9. Did the patient have a similar reaction to the same or similar drugs in any previous exposure?	+1	0	0	0	
10. Was the adverse event confirmed by any objective evidence?	+1	0	0	1	Biopsy compatible with AIH

Total score: **4** → **Possible adverse drug reaction**

Case 46 by: ***Ferronato et al***	Yes	No	Do not know	Score	Comment
1. Are there previous conclusive reports on this reaction?	+1	0	0	1	≥2 published reports in which the adverse reaction has been described in detail
2. Did the adverse event appear after the suspected drug was administered?	+2	-1	0	2	Adverse reaction appeared soon after vaccination
3. Did the adverse reaction improve when the drug was discontinued, or a specific antagonist was administered?	+1	0	0	0	Spontaneous resolution
4. Did the adverse reaction reappear when the drug was readministered?	+2	-1	0	0	
5. Are there alternative causes (other than the drug) that could on their own have caused the reaction?	-1	+2	0	2	No known agent or condition that could lead to AIH
6. Did the reaction appeared when a placebo was given?	-1	+1	0	0	
7. Was the drug detected in the blood (or other body fluids) in concentrations known to be toxic?	+1	0	0	0	
8. Was the reaction more severe when the dose was increased, or less severe when dose decreased?	+1	0	0	0	
9. Did the patient have a similar reaction to the same or similar drugs in any previous exposure?	+1	0	0	0	
10. Was the adverse event confirmed by any objective evidence?	+1	0	0	1	Biopsy compatible with AIH

Total score: **6** → **Probable adverse drug reaction**

Case 47 by: ***Ferronato et al***	Yes	No	Do not know	Score	Comment
1. Are there previous conclusive reports on this reaction?	+1	0	0	1	≥2 published reports in which the adverse reaction has been described in detail
2. Did the adverse event appear after the suspected drug was administered?	+2	-1	0	2	Adverse reaction appeared soon after vaccination
3. Did the adverse reaction improve when the drug was discontinued, or a specific antagonist was administered?	+1	0	0	0	Patient declined corticosteroids. Symptoms persist.
4. Did the adverse reaction reappear when the drug was readministered?	+2	-1	0	0	
5. Are there alternative causes (other than the drug) that could on their own have caused the reaction?	-1	+2	0	2	No known agent or condition that could lead to AIH
6. Did the reaction appeared when a placebo was given?	-1	+1	0	0	
7. Was the drug detected in the blood (or other body fluids) in concentrations known to be toxic?	+1	0	0	0	
8. Was the reaction more severe when the dose was increased, or less severe when dose decreased?	+1	0	0	0	
9. Did the patient have a similar reaction to the same or similar drugs in any previous exposure?	+1	0	0	0	
10. Was the adverse event confirmed by any objective evidence?	+1	0	0	1	Biopsy compatible with AIH

Total score: **6** → **Probable adverse drug reaction**

Case 48 by: ***Ferronato et al***	Yes	No	Do not know	Score	Comment
1. Are there previous conclusive reports on this reaction?	+1	0	0	1	≥2 published reports in which the adverse reaction has been described in detail
2. Did the adverse event appear after the suspected drug was administered?	+2	-1	0	2	Flare of transaminases elevation appeared soon after vaccination
3. Did the adverse reaction improve when the drug was discontinued, or a specific antagonist was administered?	+1	0	0	1	Resolution with corticosteroids.
4. Did the adverse reaction reappear when the drug was readministered?	+2	-1	0	0	
5. Are there alternative causes (other than the drug) that could on their own have caused the reaction?	-1	+2	0	2	Known AIH, but symptoms flare with vaccination
6. Did the reaction appeared when a placebo was given?	-1	+1	0	0	
7. Was the drug detected in the blood (or other body fluids) in concentrations known to be toxic?	+1	0	0	0	
8. Was the reaction more severe when the dose was increased, or less severe when dose decreased?	+1	0	0	1	Flare was more severe after 2nd vaccine dose
9. Did the patient have a similar reaction to the same or similar drugs in any previous exposure?	+1	0	0	0	
10. Was the adverse event confirmed by any objective evidence?	+1	0	0	1	Biopsy compatible with AIH

Total score: **8** → **Probable adverse drug reaction**

Case 49 by: ***Romero-Salazar et al***	Yes	No	Do not know	Score	Comment
1. Are there previous conclusive reports on this reaction?	+1	0	0	1	≥2 published reports in which the adverse reaction has been described in detail
2. Did the adverse event appear after the suspected drug was administered?	+2	-1	0	2	Adverse reaction appeared soon after vaccination
3. Did the adverse reaction improve when the drug was discontinued, or a specific antagonist was administered?	+1	0	0	1	Resolution with corticosteroids.
4. Did the adverse reaction reappear when the drug was readministered?	+2	-1	0	0	
5. Are there alternative causes (other than the drug) that could on their own have caused the reaction?	-1	+2	0	2	Patient with PBC, but liver biopsy was typical for AIH
6. Did the reaction appeared when a placebo was given?	-1	+1	0	0	
7. Was the drug detected in the blood (or other body fluids) in concentrations known to be toxic?	+1	0	0	0	
8. Was the reaction more severe when the dose was increased, or less severe when dose decreased?	+1	0	0	0	
9. Did the patient have a similar reaction to the same or similar drugs in any previous exposure?	+1	0	0	0	
10. Was the adverse event confirmed by any objective evidence?	+1	0	0	1	Biopsy compatible with AIH

**Case 50 by:** ***Mahalingham et al***	Yes	No	Do not know	Score	Comment
1. Are there previous conclusive reports on this reaction?	+1	0	0	1	≥2 published reports in which the adverse reaction has been described in detail
2. Did the adverse event appear after the suspected drug was administered?	+2	-1	0	2	Adverse reaction appeared soon after vaccination
3. Did the adverse reaction improve when the drug was discontinued, or a specific antagonist was administered?	+1	0	0	1	Resolution with immunosuppression.
4. Did the adverse reaction reappear when the drug was readministered?	+2	-1	0	0	
5. Are there alternative causes (other than the drug) that could on their own have caused the reaction?	-1	+2	0	2	Patient with liver transplantation due to AIH, but no evidence of active disease before vaccination.
6. Did the reaction appeared when a placebo was given?	-1	+1	0	0	
7. Was the drug detected in the blood (or other body fluids) in concentrations known to be toxic?	+1	0	0	0	
8. Was the reaction more severe when the dose was increased, or less severe when dose decreased?	+1	0	0	0	
9. Did the patient have a similar reaction to the same or similar drugs in any previous exposure?	+1	0	0	0	
10. Was the adverse event confirmed by any objective evidence?	+1	0	0	1	Biopsy compatible with AIH

Total score: **7** → **Probable adverse drug reaction**

**Case 51 by:** ***Lee et al***	Yes	No	Do not know	Score	Comment
1. Are there previous conclusive reports on this reaction?	+1	0	0	1	≥2 published reports in which the adverse reaction has been described in detail
2. Did the adverse event appear after the suspected drug was administered?	+2	-1	0	2	Adverse reaction appeared soon after vaccination
3. Did the adverse reaction improve when the drug was discontinued, or a specific antagonist was administered?	+1	0	0	0	Resolution without need for immunomodulation.
4. Did the adverse reaction reappear when the drug was readministered?	+2	-1	0	0	
5. Are there alternative causes (other than the drug) that could on their own have caused the reaction?	-1	+2	0	2	No known agent or condition that could lead to AIH
6. Did the reaction appeared when a placebo was given?	-1	+1	0	0	
7. Was the drug detected in the blood (or other body fluids) in concentrations known to be toxic?	+1	0	0	0	
8. Was the reaction more severe when the dose was increased, or less severe when dose decreased?	+1	0	0	0	
9. Did the patient have a similar reaction to the same or similar drugs in any previous exposure?	+1	0	0	0	
10. Was the adverse event confirmed by any objective evidence?	+1	0	0	1	Biopsy typical for AIH

Total score: **6** → **Probable adverse drug reaction**
